# A UK Perspective on Pain and Atypical Performance - When the Maths doesn’t Add up!

**DOI:** 10.1192/j.eurpsy.2022.157

**Published:** 2022-09-01

**Authors:** V. Hall

**Affiliations:** NeuroMind Works, Director, Sutton Coldfield, United Kingdom

**Keywords:** performance validity, symptom validity, malingering, pain

## Abstract

This presentation provides an overview of factors that can cause symptom exaggeration and/or fabrication in chronic pain. It will explore how symptom and performance validity tests can be applied to chronic pain in the context of a malingering framework and the problems of implementing this in the UK through a case example.

**Disclosure:**

No significant relationships.

